# Retraction: Seizure freedom without seizure medication following stereoelectroencephalography implantation: a case report of drug-resistant post-traumatic epilepsy

**DOI:** 10.3389/fneur.2024.1502288

**Published:** 2024-10-04

**Authors:** Frontiers Editorial Office

The journal retracts the article cited above.

Following publication, the authors contacted the journal to inform about the duplicate publication of the article cited above in Frontiers in Neurology and Neurological Case Reports:

Tran, A., and Bunch, M. (2024). Seizure freedom without seizure medication following stereoelectroencephalography implantation: A case report of drug-resistant post-traumatic epilepsy. *Neurology Case Reports* 7(1), 1044.

An investigation was conducted in accordance with Frontiers' policies, whereby it was confirmed that the authors submitted the manuscript for consideration in two journals simultaneously.

Following the Committee on Publication Ethics guidelines, this article, published most recently, is retracted.

This retraction was approved by the Chief Executive Editor of Frontiers. The authors agree to this retraction.

